# History and scientific background on the economics of abortion

**DOI:** 10.1371/journal.pone.0257360

**Published:** 2021-09-13

**Authors:** Brittany Moore, Yana van der Meulen Rodgers, Ernestina Coast, Samantha R. Lattof, Cheri Poss

**Affiliations:** 1 Ipas, Chapel Hill, North Carolina, United States of America; 2 Department of Labor Studies and Employment Relations, Department of Women’s and Gender Studies, Rutgers University, Piscataway, New Jersey, United States of America; 3 Department of International Development, London School of Economics and Political Science, London, United Kingdom; PLOS, UNITED KINGDOM

## Abstract

**Background:**

Approximately one quarter of all pregnancies globally end in abortion, making it one of the most common gynecological practices worldwide. Despite the high incidence of abortion around the globe, the synthesis of known economic outcomes of abortion care and policies is lacking. Using data from a systematic scoping review, we synthesized the literature on the economics of abortion at the microeconomic, mesoeconomic, and mesoeconomic levels and presented the results in a collection of studies. This article describes the history and scientific background for collection, presents the scoping review framework, and discusses the value of this knowledge base.

**Methods and findings:**

We conducted a scoping review using the PRISMA extension for Scoping Reviews. Studies reporting on qualitative and/or quantitative data from any world region were considered. For inclusion, studies must have examined one of the following outcomes: costs, impacts, benefits, and/or value of abortion-related care or policies. Our searches yielded 19,653 unique items, of which 365 items were included in our final inventory. Studies most often reported costs (n = 262), followed by impacts (n = 140), benefits (n = 58), and values (n = 40). Approximately one quarter (89/365) of studies contained information on the secondary outcome on stigma. Economic factors can lead to a delay in abortion care-seeking and can restrict health systems from adequately meeting the demand for abortion services. Provision of post-abortion care (PAC) services requires more resources then safe abortion services. Lack of insurance or public funding for abortion services can increase the cost of services and the overall economic impact on individuals both seeking and providing care.

**Conclusions:**

Consistent economic themes emerge from research on abortion, though evidence gaps remain that need to be addressed through more standardized methods and consideration to framing of abortion issues in economics terms. Given the highly charged political nature of abortion around the world, it is imperative that researchers continue to build the evidence base on economic outcomes of abortion services and regulations.

## Introduction

Approximately 73.3 million induced abortions occur annually [1], and approximately half of those are unsafe [2]. Globally, abortion rates have fallen somewhat over time, from 40 to 39 abortions per 1000 women of reproductive age between 1990–1994 and 2015–2019. Most of this decline occurred in developed countries. Despite the overall decline in abortion rates, the absolute number of abortions considered unsafe (according to standards set by the World Health Organization) has remained high, with close to 7 million women in developing countries receiving treatment for abortion complications. In Latin America and Sub-Saharan Africa alone, where abortion access is highly restricted, three quarters of all abortions are unsafe [3].

These numbers clearly demonstrate the stark differences in access to safe abortion services around the world. Individuals seeking abortion services can face considerable barriers to care, including (but not limited to) long travel distances to facilities, stigma, lack of partner or family support, and limited economic resources. As seen more recently, the COVID-19 pandemic has exacerbated many of the existing socio-economic inequalities, including the disproportionate burdens of poverty and violence on women. Recent estimates by the Guttmacher Institute predict an enormous impact of pandemic-related challenges on reproductive outcomes, including 15 million additional unintended pregnancies and three million additional unsafe abortions globally [4]. COVID-19’s introduction into a landscape of abortion restrictions in many countries has intensified the barriers that providers and communities already face, with disproportionate impacts on low-income abortion seekers. At the same time, the pandemic has forced abortion providers to think of novel ways to service patients that allow for social distancing, protection of workers and patients, and adherence to the regional restrictions on abortion care.

Abortion care is changing [5], whether due to the increased use of pharmaceuticals to induce abortion [6] or the increased access and use of telehealth. At the same time, national laws, protocols, and funding provisions have shifted in various directions to either support or restrict access to care [7]. Despite the attention given to abortion access and abortion laws by governments, the media, and civil society, the economic outcomes of abortion services and abortion policies are poorly documented [8]. Policymakers and advocates have access to relatively little systematic evidence on the economic outcomes of abortion [9]. Clear, synthesized economic information on the economic outcomes of abortion services and policies can be a useful source of information to aid in decision-making for governments, policymakers, advocates, and other key stakeholders. Much of the existing information at hand consists of direct costs of abortion care services while fewer studies consider the indirect costs and economic impact of seeking abortion care or the far-reaching economic outcomes of abortion policies on the individual, community, and national level. Although some of these outcomes, such as lost wages over time and missed educational opportunities, may not be easily measured, they are still highly relevant to making fully informed decisions on abortion care and policies.

To address these critical gaps, we conducted an extensive scoping review that systematically scoped existing social science literature on the economics of abortion, specifically the impact of abortion care (including un/safe abortion and post-abortion care) and abortion policies on economic outcomes. Our review synthesizes the evidence base and identifies evidence gaps on the costs, impacts, and benefits of abortion to stakeholders at three different economic levels: microeconomic (abortion seekers and their households) [10], mesoeconomic (communities and health systems) [11], and macroeconomic (societies and nation states) [12]. Detailed results from these three analyses as well as the links between the economics of abortion and stigma are presented in separate companion articles [10–13]. The objective of this article is to introduce the collection by discussing the relevant history and scientific background on the economics of abortion, the framework of our systematic review, and how this collection could benefit the field.

## History and scientific background

Abortion practices and laws restricting them have existed for centuries. Although people from around the world have been regulating their fertility through various means, the Egyptians were the first to document various abortion techniques around 1550 BCE [14]. These techniques were predominantly non-surgical and centered around the use of herbs. By the early 1800s, abortion practices had modernized and included surgical procedures with appropriate sanitation and anesthesia. Laws that criminalized abortion also appeared during this period, which in various countries could be punishable by the death penalty. The United States was not far behind in passing anti-abortion legislation, but the punishment was generally less severe [15]. By the mid-1800s, abortions were being performed fairly frequently in countries around the globe, and this remains true through the current period.

Despite this historical precedent and global prevalence, abortion seekers still have very different experiences with abortion around the world. Pregnant people in higher-income economies generally have greater access to safe abortions, while pregnant people in lower-income countries experience greater health risks in getting abortions due to greater resource-based obstacles to care (i.e., funds to reach point of access), less equipped healthcare infrastructure as well as abortion laws that restrict access to safe abortions [1]. Abortion laws have a long history that has often been shaped by deeply entrenched religious views, political ideologies, and patriarchal structures. These ideologies in turn are closely intertwined with stigmas around abortion in which women who seek one are viewed as straying from feminine ideals that include women’s natural fecundity, the irrevocability of their roles as mothers, and their instinctive nurturance of those who need care [16].

Abortion stigma can contribute to the creation and perpetuation of restrictive abortion legislation, which can have the likely unintended consequence of increasing, rather than decreasing, abortion rates. There is no definitive evidence that legal restrictions on abortions result in fewer abortions. In fact, findings in Sedgh *et al*. [17] show no association between abortion rates across countries and the legal status of abortion in those countries. If anything, countries with more restrictive abortion policies have more unsafe abortions, and countries that legalize abortions see a shift from clandestine, unsafe abortions to legal, safe abortions without an increase in overall abortion rates. Legalizing abortion is seen by a growing number of multilateral agencies, non-governmental organizations, scholars, and advocates as a necessary step toward reducing unsafe abortions and improving women’s reproductive health. Yet despite this authoritative shared view that access to safe, legal abortion is a fundamental right for women, more than 60 countries still ban abortion completely or only permit it to save the woman’s life.

The widespread dissemination of information through the internet has helped to destigmatize both abortion and contraception, and it has provided healthcare practitioners and women with clinical information about fertility control and safe abortion procedures, including abortion pills, otherwise known as medical abortion [18]. The World Health Organization approved the combination of mifepristone and misoprostol pills to induce abortion in the first twelve weeks of pregnancy and it placed the drugs on its list of essential medicines in 2005. However, availability and access to misoprostol and mifepristone is shaped by contextual health systems and regulatory frameworks. This accounts for the wide variation in access, availability and costs across geographies, legalities and social contexts [19]. The use of these drugs to perform medical abortions is still not a widely available option in lower-income countries. High prices and restrictive regulations, especially in the case of mifepristone, has been one of the limiting factors in the widespread use of medical abortions. Inequalities in access to both medications at affordable costs is also shaped by the global variations in essential medicines lists, with only 50 of 158 countries analyzed in the Global Abortion Policies Database including both misoprostol and mifepristone, not necessarily for abortion-related care [20]. By 2011, misoprostol was approved in over 80 countries, mostly for the prevention and treatment of gastric ulcers. However, mifepristone was only approved explicitly for abortion in 45 countries, mostly higher-income countries [21].

Considering the evidence, it is clear that there is a broad range of economic impacts and outcomes related to abortion access. However, despite the high incidence of abortion around the globe, we lack synthesis of the known economic costs and outcomes–at a variety of scales–of abortion care and abortion policies. Hence the economic consequences of abortion and policies affecting abortion provision are poorly understood. This evidence gap motivated our scoping review, the framework for which is discussed in the next section.

## Framework of the scoping review

Our study reviews existing evidence on the economics of abortion and conceptualizes important issues around abortion, especially the costs, benefits, and impacts of abortion. The analysis synthesizes the evidence base and identifies evidence gaps on the costs and benefits of abortion to stakeholders at three different economic levels: micro (abortion seekers and their households), meso (communities and health systems) and macro (societies and nation states).

At the micro-level, we provide a comprehensive examination of individual’s decision making around contraceptive use, fertility, and abortion. The framework is based on a set of economics tools related to costs and benefits that model preferences and behaviors around fertility and abortion. At the meso-level, we consider the costs and benefits of abortion services in the context in which they take place, particularly communities and medical systems. Finally, at the macro-level, the project explores how access to abortion services and changes in abortion laws affect broad aggregates such as women’s labor supply, educational attainment, indicators of societal wellbeing such as crime, and overall gross domestic product (GDP).

Our scoping review of the economics of abortion is based on micro foundations and particularly, on an economic model of fertility that includes the cost of contraception and abortion [8, 22]. We use the following production function to represent the determinants of fertility:

F=f(C,A,ε)

In this equation, a person’s actual fertility depends on the use of two methods to control fertility: contraceptive methods *C* and induced abortion *A*. Actual fertility also depends on an idiosyncratic element represented by the term ε, which includes both random chance and natural fecundity (assumed to be a given and not changeable by the person’s actions). These two methods of birth control each have an inverse relationship with fertility. The use of contraceptives and induced abortion both depend on the pecuniary and time costs of accessing them, while ignoring either method is assumed to be free and to involve no time costs. Both options also involve “utility costs,” which encompass social, religious, philosophical, and institutional factors related to birth control and abortion. The key decision in this model is for a person to choose the appropriate levels of *C* and/or *A* that will prevent actual fertility *F* from exceeding desired fertility F*, subject to the constraints the person faces regarding the monetary, time, and utility costs of both methods.

As one example of a utility cost that could influence an individual’s decision making about fertility, a person might live in a country in which abortion is very common and socially accepted because historically modern methods of contraception were less available. In contrast, someone could live in a country in which abortion is illegal and highly stigmatized. An abortion seeker might live in a country where they can buy abortion pills on the black market or through the internet. An abortion seeker might live in a country with a shortage of trained medical personnel and sterile facilities, making it difficult to get a safe abortion. What might happen if the cost of contraception or abortion rises? This model traces the effect of such an increase, which could feasibly arise should local healthcare providers experience funding cuts or an increase in the price of supplies. These changes are expected to impact individuals’ contraceptive usage, abortion, and actual fertility in ways that vary by their ability to absorb the higher costs, as well as the norms, beliefs, and institutional constraints associated with fertility control. Such changes may also increase the time that people need to find a reproductive healthcare provider. The extent to which individuals re-optimize their decisions around *C* and *A* depends not only on their ability to pay for *C* and *A* but also on the utility costs of these reproductive healthcare services and the form of the production function (*f*).

At the microeconomic level, this framework bolsters our focus on the financial cost of an abortion in the scoping review. Because the production function terms each include “utility costs”, our scoping review also focused on terms closely related to factors affecting the utility costs of abortion, and especially the economic impact, benefits, and values. At the mesoeconomic level, we aggregate up from these micro foundations to the level of communities and health systems. In particular, the provision of abortion care services requires financial and physical resources on the part of health facilities, and health providers have incentives to minimize their costs while maintaining or even improving the quality of abortion care services. At the macroeconomic level, we aggregate up from the micro model to the level of societies and nation states. People’s decision making around their fertility has direct repercussions for outcomes such as their education and employment, which in turn can affect macroeconomic aggregates such as labor supply and even GDP growth. More broadly, institutional factors associated with the model’s “utility costs” include government policies around the legality of abortion as well as public sector financing of abortion services. National laws around abortion are thus another important dimension of the model that can impact individual abortion seekers at the micro level, medical systems at the meso level, and human capital outcomes at the macro level.

It is critical to think of and beyond the direct costs of abortion services and individual economic burden of abortion policies to include the individual, family, community, health systems, national, and international level economic impacts. Rarely does the restrictive impact of an abortion policy take place in a vacuum nor does the cost of an abortion service only impact one day out of an individual’s life. To further define the economic outcomes of abortion services, our scoping review addresses current knowledge on the economics of abortion as well as the evidence gaps. This work articulates the costs, benefits, and value of abortion to women, households, communities, health systems, and societies with a traditional economic lens. It will also define and enumerate the non-traditional economic value, opportunity costs, and beneficiaries of abortion.

As abortion-related decisions and policies have their own unique economic implications, it is important to capture the full range of cost and health impacts of the services as well as the intangible factors not typically articulated as part of the direct economic impact of abortion, which may include: the impact of social exclusion, the educational and professional opportunities lost to women due to unintended pregnancy, the costs and economic impact of being denied abortion services, and longer term social and health effects of unsafe abortion. Traditional economic approaches tend to look at costs from the payer perspective and benefits from the user perspective. This systematic review seeks to broaden this lens for a full, comprehensive look at the beneficiaries beyond direct users and the health systems to include the implications of discrimination and stigma associated with abortion.

Ultimately, a scoping review on the evidence for the value, impact, and costs of abortion will help the field better understand our current programmatic functions. It would also be a valuable tool to make the case for how abortion is prioritized and addressed as a public health issue by governments and donors, and to help to inform our strategic priorities for future interventions.

## Economics of abortion: The collection

### Methods

We took a systematic approach to finding evidence on the economics of abortion by conducting a scoping review of relevant literature. We developed a protocol [23] following the Preferred Reporting Items for Systematic review and Meta Analyses (PRISMA) extension for Scoping Reviews (PRISMA-ScR) and reporting guidelines [24] to ensure our review was manageable, transparent, and reproducible. Since we were interested in analyzing the available, known evidence on the economic outcomes of abortion care and abortion policies, and we expected to locate a broad and diverse body of evidence on this topic [25], we chose to conduct a scoping review instead of a systematic review.

The scoping review considered any peer-reviewed journal article on induced abortion and/or post-abortion care from any world region [23]. Items also must have been published in English, French, Spanish, Dutch, or German from 1 September 1994 to 15 January 2019. The beginning point marks the start of the International Conference on Population and Development in Cairo, Egypt. This is an important date due to the resulting changes in the global discourse following this conference, shifting from a focus mostly on family planning to a broader continuum of care around sexual and reproductive health and rights.

The articles in this scoping review must have qualitative and/or quantitative data on abortion care or abortion policies at the microeconomic, mesoeconomic, or macroeconomic levels. The articles also must have included information on at least one of the following four types of economic outcomes: financial cost (cost of receiving or providing abortion care or financial costs resulting from abortion policies); impact (the effect or influence of abortion care or abortion policies); benefit (advantages or profits gained from receiving or providing abortion care or implementing abortion policies); and value (the importance, worth, welfare gains, or utility of receiving or providing abortion care or implementing abortion policies) [23]. Additional details on the inclusion and exclusion criteria for this scoping review, testing the search terms, and the process to extract the data can be found in the published scoping review protocol [23].

Findings in the economics of abortion collection are reported using a systematic narrative synthesis framework in which the results are presented narratively and organized thematically, supplemented with tables of descriptive statistics on included studies and their outcomes. In nearly all of the studies that we reviewed, authors tend to refer to study participants as ‘women’ or ‘girls’. We have used inclusive and/or gender-neutral language in our own writing in an effort to be more inclusive of transgender and nonbinary individuals. However, in the methods and results sections of the articles in the economics of abortion collection, we used language that is reflective of the terminology used by authors to describe study participants in the referenced articles.

### Composition of articles in the collection

At the microeconomic level, we synthesized data from 230 studies on abortion care and policies [10]. Individual-level costs of abortion-related care around the globe have implications for the timing and type of care sought. To pay for abortion services, some people can forgo other expenditures or be pushed further into poverty and/or debt. Economic factors influence the time it takes to reach and/or receive abortion services and can lead to significant delays in care-seeking, which in turn impact the type of care sought, the gestational age at which care is obtained, and the cost of service.

At the mesoeconomic level, we synthesized data from 150 studies on abortion care and policies [11]. The evidence most frequently examines abortion costs to health systems and health facilities, particularly in high-income countries. The provision of post-abortion care services requires a disproportionate amount of financial and physical resources. Health facilities and health systems can realize financial savings while maintaining or even improving quality of abortion care services. Analyzing and comparing the costs of providing abortion services globally can be challenging due to variation across studies in identifying components of care and documenting costs.

At the macroeconomic level, we synthesized data from 158 studies on abortion care and policies, with much of the evidence focused on costs at the national level [12]. Public sector coverage of abortion costs is limited and inconsistent around the world. Evidence shows that removing restrictive abortion laws can have positive effects for education and labor, though the political economy around abortion legislation and its impacts are complicated and controversial.

In this collection, we also sought to better understand the intersections between abortion stigma and economic outcomes [13]. Out of the 89 articles with abortion stigma data, only 32 studies included stigma findings directly tied to the primary economic outcomes. In the remaining articles, stigma is mentioned in terms of its effects on the context or research methods but is less directly related to the primary economic outcomes. Abortion stigma can serve as a barrier to prevent individuals from obtaining correct information regarding abortion services and laws, leading to unnecessary increases in costs of care and significant delays. Cost of abortion services can be substantial, and individuals who are unable to disclose to and/or rely on their social support network are less likely to have adequate financial resources to access abortion services.

## Conclusion

The onset of a global pandemic into a restrictive abortion environment in many countries around the globe makes it vital to conduct research on novel approaches and access to abortion care that will meet the needs of individuals facing different structural barriers, political obstacles, and socioeconomic backgrounds as they attempt to manage their fertility. COVID-19 has also forced abortion providers to devise new ways to manage healthcare with the implementation of protocols, including telemedicine, that satisfy the constraints of legislative restrictions and the social realities of the COVID-19 pandemic. Hence there is a clear need for more research on people’s needs for abortion services, the best potential methods of providing abortion services in this landscape of constraints, and the systems needed to help people access abortion without shame. However, this research cannot be done in a vacuum, making it all the more important to have a readily available body of knowledge on the economic costs and outcomes of abortion services and policies from the past few decades.

Although relatively few studies are defined explicitly by their authors or their methodology as “economic” studies, our review shows that there is a wealth of economically relevant information that can be gleaned from the evidence base. For example, whilst very little evidence uses the language of economic values and benefits of abortion, it is possible to infer micro-level values and benefits of abortion-related care by examining people’s reasons for abortion. These reasons are rarely singular; abortion-related decision-making is often the result of a complex interplay of factors (wealth, education, status, education, relationship). Moreover, the interplays between economics and delays to abortion-related care are striking. Across diverse contexts and populations, economic factors influence delays to abortion-related decision-making, attempts to seek care and the receipt of care. By unpacking the points at which economic factors introduce or compound delays to abortion-related care, greater insight into the points at which information and services might be better designed to reduce delays can be achieved. By further unpacking the intersectionality of these economic factors, we can better understand the ways in which health systems and contexts reproduce injustices and inequities.
